# Hydrothermally Modified Defatted Coconut Fiber as a Functional Fat Replacer in Reduced-Fat Cookies: A Structure-Function Study

**DOI:** 10.3390/foods15030424

**Published:** 2026-01-24

**Authors:** Patcharanun Suksangpanomrung, Pitiporn Ritthiruangdej, Nantawan Therdthai, Arisara Hiriotappa

**Affiliations:** Department of Product Development, Faculty of Agro-Industry, Kasetsart University, Bangkok 10900, Thailandfaginwt@ku.ac.th (N.T.);

**Keywords:** agro-industrial by-product, lignocellulosic biomass, hydration properties, dough viscoelasticity, modification techniques, nutritional reformulation, circular economy

## Abstract

This study investigated the combined influence of hydrothermal treatment and particle size on the techno-functional properties of defatted coconut residue (DCR) to optimize its use as a hydrocolloid fat replacer. A 3 × 2 factorial design evaluated boiling and autoclaving treatments in combination with coarse and fine milling. Fine particle fractions (boiling-fine [BF] and autoclaved-fine [AF]) were identified as optimal, exhibiting peak water-holding capacity (WHC) (10.95 g/g) and oil-holding capacity (4.57 g/g) due to maximized surface area and thermal unblocking of capillary networks. When incorporated into cookies, all DCR formulations qualified as “reduced-fat” (30% reduction) and “high-fiber” (6 g/100 g) products. Crucially, the extreme WHC of fine fractions induced severe water competition within the dough, leading to a direct inverse correlation with quality, characterized by a restricted spread ratio (6.9) and increased hardness (27 N). Furthermore, thermal leaching of Maillard precursors suppressed excessive browning, improving cookie color. While the BF fraction provided the best functional balance, future research should optimize dough moisture to mitigate the impact of high fiber hydration on texture. These findings demonstrate DCR’s potential for agro-food valorization and improved human health.

## 1. Introduction

The global food industry is increasingly shifting towards a circular economy (CE) model, driven by the need to address resource scarcity, reduce the substantial environmental impact of agri-food waste, and align with global Sustainable Development Goals (SDGs) [1,2,3]. This transition emphasizes the valorization of processing by-products, converting materials traditionally discarded into high-value functional ingredients [4,5]. Beyond sustainability, the incorporation of functional fibers into daily diets directly impacts human health by enabling the development of reduced-fat products, which are essential for mitigating the global prevalence of obesity and related non-communicable diseases (NCDs).

Global coconut production was estimated at 62.41 million metric tonnes in 2022, with leading producers including Indonesia, the Philippines, and India [6,7]. This industry generates approximately 61.5 million tons of coconut waste globally, with a projected annual growth rate of 1% [7]. Approximately 60–65% of each coconut fruit processed is considered waste, primarily husks and shells [6,8]. The accumulation of these residues leads to significant environmental issues, such as unpleasant odors, landfill clogging, and greenhouse gas emissions [9]. Furthermore, the high moisture content in these by-products (exceeding 80%) accelerates microbiological growth and spoilage, necessitating immediate processing to ensure safety and nutritional value [10,11]. Currently, coconut by-products, such as defatted coconut flour, are often underutilized or discarded as low-value animal feed, resulting in the waste of biological resources [12,13].

Coconut residue (or spent coconut meal), a significant by-product of the coconut milk and oil extraction industries, represents a promising candidate for this valorization approach [14,15]. Defatted coconut residue (DCR) is renowned for its exceptional nutritional profile, particularly its high total dietary fiber (TDF) content, which often exceeds 60% [16,17]. This fiber is predominantly insoluble dietary fiber (IDF), which imparts significant swelling and adsorption capacities, making DCR an ideal, clean-label ingredient for food and pharmaceutical applications [18,19,20]. Despite its high value, DCR remains significantly underutilized, often ending up as low-value animal feed or waste, underscoring the need for robust processing techniques to maximize the functional potential of its fiber and protein fractions [15].

To unlock the full hydrocolloid functional potential of lignocellulosic materials like DCR, strategic modification of the fiber structure is essential, particularly to enhance key techno-functional properties such as water-holding capacity (WHC) and oil-holding capacity (OHC) [21]. Among various processing methods, hydrothermal modification (HTM), including boiling and autoclaving, represents an industrially feasible, non-chemical approach for structural refinement [22,23]. HTM utilizes heat and moisture to disrupt the tightly bound fiber matrix. The intense conditions of autoclaving (121 °C, 15 psi) are known to induce partial depolymerization of polysaccharides through the cleavage of glycosidic linkages, which significantly reduces solution viscosity but often leads to more rigid gels and increased gelling ability in specific food matrices due to changes in molecular entanglements and aggregation [24,25,26,27]. However, the thermal intensity is critical, as excessive heat can lead to structural collapse or the formation of degradation products [28]. Crucially, the effect is highly matrix-dependent: while HTM can increase viscosity in dilute suspensions, it may decrease it in dough matrices due to protein denaturation and starch effects [29]. Furthermore, physical manipulation via particle size reduction is a critical cofactor: decreasing particle size generally increases specific surface area, which often enhances OHC [30,31]. However, the effect on WHC is usually a delicate balance between maximizing surface area and preserving structural integrity and macro-porosity during milling [32,33,34].

Fat plays a multi-functional role in cookie systems, acting as a lubricant that contributes to dough plasticity, machinability, and final texture [35,36]. During mixing, fat coats flour particles, forming a barrier that prevents water absorption by gluten proteins and thereby inhibits gluten development [37,38]. This mechanism ensures a ‘short’ dough with a tender, crumbly, and melt-in-the-mouth texture [39,40]. Conversely, fat reduction leads to less coating of flour particles, allowing for more extensive gluten formation, which results in a tougher, more complex texture and a decreased spread ratio [41,42]. The distribution and type of fat are therefore critical for maintaining the desired quality and structural integrity of reduced-fat cookies [43,44].

The increasing consumer demand for healthier baked goods has accelerated research into replacing saturated fats with carbohydrate-based alternatives [45,46]. In cookies, fat is crucial for creating the desirable soft texture by limiting gluten network formation and controlling cookie spread during baking [47,48]. Consequently, reducing fat content often results in undesirable structural changes, specifically increased dough hardness and reduced spread ratio [49,50]. The incorporation of high-WHC fibers increases the elastic and viscous moduli (G’ and G”) of the dough, indicating enhanced rigidity and resistance to flow [51,52]. To mitigate these textural defects, a successful fat replacer must possess high WHC and OHC to mimic the lubricating functions of fat [53,54]. The hydrophilic nature of these hydrocolloids promotes water competition, disrupting the dough’s overall viscoelastic properties [52,55,56].

Dietary fibers sourced from by-products, such as wheat bran and fruit pomace, are highly effective in this role [27,57]. DCR, with its porous structure and exceptional TDF, has strong potential as a functional fat replacer. However, the cookie quality is highly susceptible to the functional properties of the incorporated fiber [58,59]. Despite the acknowledged potential of DCR, two critical gaps remain in the literature: (1) a systematic comparative study focusing on the interaction effects of industrial-scale thermal treatments (boiling vs. autoclaving) and particle size ranges (coarse vs. fine) on DCR’s structure is lacking [60,61], and (2) there is an absence of research directly linking tailored DCR properties to the ultimate rheological behavior of cookie dough and its final physical attributes [62,63].

Therefore, this integrated study aims to systematically investigate the combined influence of hydrothermal treatment and particle size on DCR and to critically evaluate their performance as fat replacers in reduced-fat cookies. The specific objectives are to: (1) determine the combined effects of processing on the physicochemical and functional properties of DCR powder; and (2) directly correlate these properties with the dough rheology and final quality of the reduced-fat cookie.

## 2. Materials and Methods

### 2.1. Raw Materials and Sample Preparation

#### 2.1.1. Raw Materials and Chemicals

Residual grated coconut (initial moisture content: 55–66%; fat content: 14–26%) was obtained from Ampol Food Processing Co., Ltd. (Nakhon Pathom, Thailand). The raw residue was stored at 4 °C and transported to the laboratory within 2 h of collection. All chemical reagents used were of analytical grade.

#### 2.1.2. Other Ingredients for Cookie Formulation

All-purpose wheat flour (UFM Food Center Co., Ltd., Bangkok, Thailand), unsalted butter (KCG Corporation Co., Ltd., Samut Prakan, Thailand), and icing sugar (Thai Roong Rueang Industry Co., Ltd., Bangkok, Thailand) were sourced locally. Flavoring agents included liquid vanilla (McCormick & Company, Inc., Hunt Valley, MD, USA), liquid coconut, and liquid butter (KH Roberts, Samut Prakan, Thailand).

### 2.2. Defatted Coconut Residue (DCR) Powder Preparation

**Figure 1:**
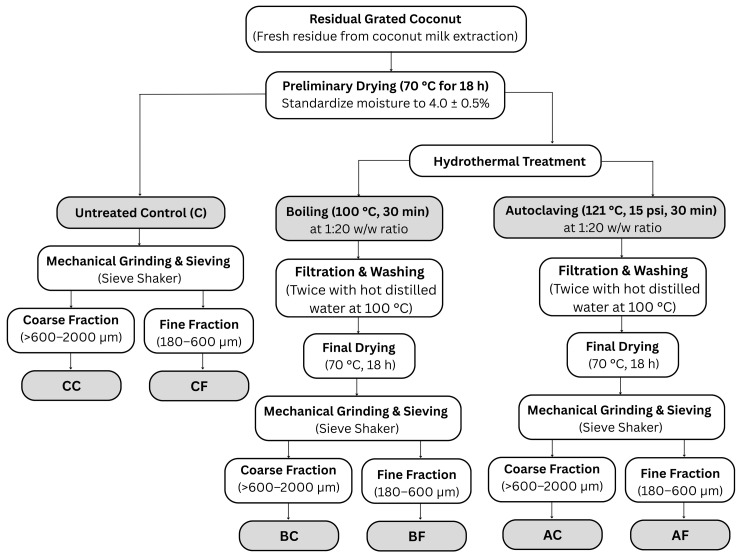
Schematic flowchart illustrating the preparation process of defatted coconut residue (DCR) powder, including hydrothermal treatments (boiling and autoclaving) and particle size fractionation.

The study utilized a 3 × 2 factorial arrangement combining three hydrothermal defatting methods and two particle size fractions. The preparation process of DCR powders, involving hydrothermal modification and mechanical grinding, is systematically presented in Figure 1.

#### 2.2.1. Preliminary Drying and Preparation of Base Material

Prior to hydrothermal treatment, the raw coconut residue was dried in a tray dryer (BWS model, Frecon, Samut Prakan, Thailand) at 70 °C for 18 h to standardize the initial moisture content to 4.0 ± 0.5% (dry basis). The resulting dried material served as the untreated control (C) and the base for hydrothermal treatments: boiling (B) and autoclaving (A).

#### 2.2.2. Hydrothermal Defatting: Boiling Treatment (B)

The dried coconut base was mixed with distilled water at a 1:20 (*w*/*w*) ratio and boiled at 100 °C for 30 min. The mixture was cooled to 30–40 °C and frozen at −18 °C for 3 h to ensure complete solidification of the residual surface fat, as large fat globules require longer crystallization times [64,65]. The mixture was filtered, washed twice with hot distilled water (100 °C), squeezed, and dried at 70 °C for 18 h.

#### 2.2.3. Hydrothermal Defatting: Autoclaving Treatment (A)

The dried coconut base (1:20 *w*/*w* water) was sterilized in an autoclave (FnB Machinery & Solution, Bangkok, Thailand) at 121 °C and 15 psi for 30 min. After autoclaving, the sample was cooled and frozen at −18 °C for 90 min. The shorter duration was justified by greater fat emulsification or destabilization under pressure, which facilitated faster fat layer formation [66,67]. Subsequent steps (filtration, washing, and drying) followed the same procedure as the boiling treatment.

#### 2.2.4. Particle Size Fractionation

The dried residues were ground and fractionated using a sieve shaker (AS200 digit, Retsch, Haan, Germany) into coarse particles (>600–2000 µm) and fine particles (180–600 µm). The six powders were labeled CC, CF, BC, BF, AC, and AF. Particle sizes were selected to balance hydration capacity and industrial feasibility [68,69]. The coarse fraction serves as a baseline for large-particle-size impacts [68], while the fine fraction optimizes specific surface area for fiber utilization as a fat replacer [70].

### 2.3. DCR Powder Characterization

#### 2.3.1. Proximate and Dietary Fiber Composition

The proximate composition of the samples was determined using standard AOAC official methods [71]. Moisture content was analyzed using the hot-air oven method at 105 °C (AOAC 925.10). Crude fat was extracted with petroleum ether using an automated Soxhlet extraction unit (AVANI 2050, Foss, Hillerød, Sweden) following AOAC 920.39. Total nitrogen (TN) was determined by the Kjeldahl method (AOAC 979.09), and crude protein was calculated using a conversion factor of 6.25. Crude fiber content was determined according to AOAC 962.09 using a fiber analyzer (Fibertec 2010, Foss, Hillerød, Sweden). This method involved sequential digestion with 1.25% (*w*/*v*) H_2_SO_4_ and 1.25% (*w*/*v*) NaOH, followed by filtration, washing, drying, and incineration at 550 °C. Ash content was analyzed by incineration at 550 °C until a constant weight was achieved (AOAC 923.03). Total carbohydrate was calculated by difference: 100 − [moisture (%) + fat (%) + protein (%) + crude fiber (%) + ash (%)]. Insoluble dietary fiber (IDF), soluble dietary fiber (SDF), and total dietary fiber (TDF) were determined using enzymatic-gravimetric methods according to AOAC 991.42, 993.19, and 985.29, respectively.

#### 2.3.2. Functional Properties

Water holding capacity (WHC), water solubility index (WSI), and oil holding capacity (OHC) were determined following the centrifugation-based procedure described by Raghavendra et al. [16].

#### 2.3.3. Color and Microstructure

Color parameters (L*, a*, b*) were measured using a spectrophotometer (UltraScan PRO, Konica Minolta, Reston, VA, USA). The whiteness index (WI) was calculated as described by Sorde and Ananthanarayan [72]. Microstructural analysis was performed using a scanning electron microscope (SEM) (JSM-6610LV, JEOL, Tokyo, Japan) at 500× and 1000× magnification. The presented SEM images are representative of multiple observed fields (*n* > 5) to ensure microstructural reliability.

### 2.4. Cookie Preparation and Baking

A 3 × 2 factorial design was used. The reduced-fat cookie base included wheat flour (70 g), DCR powder (30 g), unsalted butter (70 g), icing sugar (36.36 g), and flavoring agents. The cookies were prepared using a standard short-dough formulation [73]. Unsalted butter and icing sugar were creamed for 3 min at speed 3 using a KitchenAid mixer (KMX 51, Kenwood, Havant, UK). Vanilla and coconut flavors were added and mixed for 1 min at speed 6. Coconut powder was incorporated (30 s at speed 1), followed by the gradual addition of sifted wheat flour (1 min). The dough was rolled to a thickness of 3 mm and cut into 38 mm diameter circles. The cookies were baked in an electric oven (E-60K, Sharp, Osaka, Japan) at 150 °C for 13 min, cooled at room temperature for 30 min, and stored in aluminum foil bags for further analysis.

### 2.5. Analysis of Cookie Quality Attributes

Proximate and dietary fiber composition were determined as described in Section 2.3.1. Physical attributes (diameter, thickness, and spread ratio) were measured with a digital caliper. The browning index (BI) was calculated using the equation cited by Bernaś and Jaworska [74] and Ayvaz et al. [75].

Dough texture was evaluated following the modified method of Park et al. [76]. Dough samples were prepared as rectangular blocks (20 mm × 20 mm × 10 mm) and subjected to a compression test using a Texture Analyzer (TA.XT plus, Stable Micro Systems, Godalming, UK) equipped with a 36 mm diameter cylindrical probe (P/36R). The test conditions were 80% strain, a pre-test speed of 2.0 mm/s, a test speed of 1.0 mm/s, and a post-test speed of 1.0 mm/s. The peak force was recorded as Hardness (N).

For the finished cookies, the 3-point bending test was performed according to Mudgil et al. [77] using a 3-point bending rig (HDP/3PB). The span between the two support base points was set at 30 mm. The probe moved with a pre-test speed of 1.5 mm/s, test speed of 2.0 mm/s, and post-test speed of 10.0 mm/s, with a trigger distance of 40 mm. The maximum force required to snap the cookie into two pieces was recorded as Hardness (N).

### 2.6. Statistical Analysis

Data were analyzed using a Two-Way Analysis of Variance (ANOVA) in SPSS (Version 29.0, IBM Corp., Armonk, NY, USA). Mean differences were separated using Tukey’s HSD post hoc test (*p* < 0.05).

## 3. Results and Discussion

### 3.1. DCR Powder Characterization

#### 3.1.1. Proximate Composition of DCR

The proximate composition of DCR powder (Table 1) confirmed that hydrothermal treatment (M), particle size (P), and their interaction (M × P) significantly affected all measured components (*p* < 0.05). Boiling and autoclaving significantly reduced crude fat content (17.80–19.87%) compared with the untreated control (24.00–26.53%). This reduction validates the efficiency of the hot-water extraction, freezing, and skimming method, which mechanically and thermally disrupts the fiber–lipid matrix to remove surface and loosely encapsulated fat [17]. Similarly, crude protein and ash content were significantly reduced (*p* < 0.05) in the treated groups. This is attributed to the thermal leaching of water-soluble components, including minerals and low-molecular-weight protein fractions, during the hydrothermal treatment and subsequent washing steps [12]. Notably, autoclaving resulted in a greater reduction in both protein and ash than boiling, suggesting that the higher temperature and pressure (121 °C, 15 psi) enhanced solubilization and the subsequent removal of these water-soluble components [78].

Conversely, crude fiber and carbohydrate content increased significantly (*p* < 0.05) after hydrothermal treatments. This shift is primarily due to the relative concentration effect, where the mass loss from the removal of non-fiber components purifies the remaining fiber fraction, increasing its proportion relative to the total dry weight [12]. The autoclaved-coarse sample (AC) achieved the highest crude fiber content (53.72%), indicating the effectiveness of intensive thermal processing in concentrating the insoluble fiber matrix.

Particle size exhibited a significant main effect on crude fat, protein, and fiber contents. Within the untreated group, the fine fraction (CF: 180–600 µm) exhibited significantly lower protein (3.54% vs. 4.60%) and fiber (36.15% vs. 46.42%) levels, but higher fat (26.53% vs. 24.00%) than the coarse fraction (CC). Mechanical shear forces during milling fracture the cellular structure, increasing the specific surface area and accelerating the leaching of water-soluble components and dispersible fiber fragments during handling [79,80]. The higher fat content in the CF fraction likely results from sample heterogeneity or from greater relative loss of fiber components, which elevates the proportion of residual fat.

#### 3.1.2. Dietary Fiber Composition of DCR

DCR powder samples were highly rich in dietary fiber, with total dietary fiber (TDF) ranging from 63.77% to 73.94% (Table 1). Hydrothermal treatment significantly increased the TDF and insoluble dietary fiber (IDF) contents compared with the untreated control (*p* < 0.001), due to the relative concentration effect described above [12]. The boiling-coarse (BC) sample achieved the highest TDF (73.94%), confirming the efficacy of hot water treatment for fiber purification.

The soluble dietary fiber (SDF) content exhibited a complex pattern. The CF fraction yielded the highest SDF (4.99%), demonstrating a mechanical solubilization effect where high shear forces physically fracture cell walls and disrupt the rigid lignocellulosic structure [19,20]. This process degrades IDF components, such as hemicellulose and cellulose, into smaller, more soluble fragments [52,81]. However, the autoclaved-coarse (AC) sample yielded the lowest SDF content (1.21%). This suggests that intensive thermal and pressure conditions likely triggered extensive molecular degradation of soluble polysaccharides [25,30] or induced chemical aggregation of soluble components into an insoluble residue [63,82]. These fragments or aggregates were subsequently lost during washing, resulting in a net loss of recoverable SDF.

#### 3.1.3. Microstructural Characteristics of DCR

**Figure 2:**
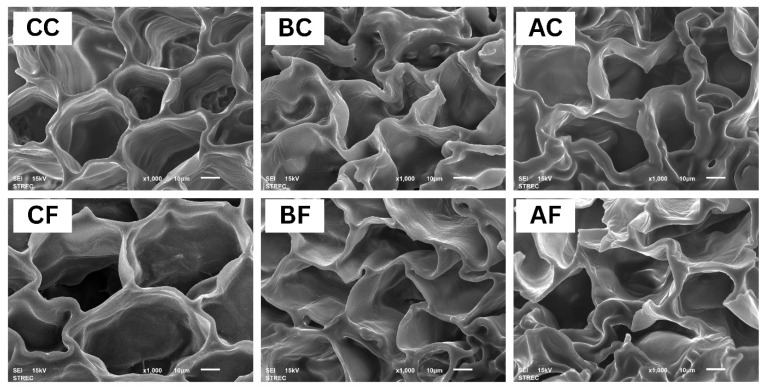
Scanning Electron Microscopy (SEM) images of Defatted Coconut Residue (DCR) powder: cross-section view at 1000× magnification, illustrating the effect of hydrothermal treatment and particle size on fiber morphology.

SEM analysis at 1000× magnification (Figure 2) revealed that all DCR samples exhibited a highly porous, layered lignocellulosic matrix consisting of hollow cellulose fibrils and distributed pores [83]. This natural porosity is directly responsible for the fiber’s substantial water-absorption capacity [83,84]. Untreated surfaces displayed a layer of residual fat coating, while boiled and autoclaved samples exhibited a significantly cleaner surface with visibly pronounced porosity and a clear honeycomb structure [16]. This structural cleaning unblocks the fiber network, explaining the increase in OHC and WHC in treated samples [85]. Boiling samples (BC and BF) maintained a more open architecture, whereas autoclaving samples (AC and AF) exhibited a more compact structure and greater distortion. This compaction aligns with the mechanism that high-pressure treatment causes greater cell wall disruption and structural collapse [3,86].

#### 3.1.4. Functional Properties (WHC, OHC, WSI) of DCR

Functional properties (Table 2) are critical predictors of performance as a fat replacer. Hydrothermal treatment significantly enhanced both WHC and OHC (*p* < 0.001), surpassing conventional fiber sources [16]. The BF fraction achieved the highest OHC (4.57 g/g) and a peak WHC (10.95 g/g). This enhancement is attributed to the synergistic effect of pore unblocking (defatting) and surface area optimization (milling) [16,85]. The fine particle size maximized the specific surface area and pore volume, enhancing physical adsorption and capillary retention [87,88]. However, the slightly lower OHC in autoclaving samples (AC, AF) suggests that excessive heat induced structural compaction, hindering the oil adsorption capacity [89]. WSI showed an inverse trend: untreated samples exhibited higher WSI than treated groups (*p* < 0.001) due to thermal leaching and the removal of soluble sugars, proteins, and minerals during washing [15,46,90], resulting in a purified, low-solubility fiber concentrate [91].

The significant interaction between the hydrothermal method and particle size (M × P) for WHC and OHC indicates that the effect of size reduction depends on the prior thermal treatment. In this study, fine particle fractions (BF and AF) exhibited peak hydration properties, attributed to the synergistic effects of pore unblocking and surface area maximization. Hydrothermal treatments effectively removed residual lipids and proteins that previously clogged the fiber’s capillary networks [14,16]. Subsequent milling into fine fractions further increased the specific surface area, exposing more hydrophilic sites for water and oil binding [92,93]. However, while decreasing particle size generally enhances WHC due to the increased surface area, excessive reduction can collapse the fiber’s porous matrix, thereby reducing the space available for water entrapment [94,95]. Our findings suggest that the 180–600 µm range provides an optimal balance, maintaining structural integrity while maximizing functional capacity [16].

#### 3.1.5. Color and Whiteness Index (WI) of DCR

The color of the DCR powder is a crucial quality indicator [96]. The CF sample was the lightest (L* = 86.78) and exhibited the highest WI (84.64), whereas the AC sample was the darkest (L* = 75.65) and exhibited the lowest WI (73.96). This darkening is attributable to the Maillard reaction, which is accelerated by the intense thermal conditions of autoclaving (121 °C, 15 psi) [97,98]. High heat promotes the condensation of reducing sugars with free amino groups, forming brown polymeric pigments (melanoidins) [19,99]. Notably, the AF sample recovered significantly more whiteness (L* = 83.78) than the AC sample. This may be explained by greater leaching of residual reducing sugars and soluble compounds from the fine fraction during washing, thereby decreasing the available substrates for the Maillard reaction.

### 3.2. Cookie Application and Quality

#### 3.2.1. Proximate and Dietary Fiber Composition of Reduced-Fat Cookies

The successful incorporation of DCR as a partial fat and flour substitute significantly altered the nutritional profile of the baked cookies (Table 3). Crucially, the primary objective of developing reduced-fat and high-fiber cookies was validated across all formulations [99,100]. All DCR formulations exhibited crude fat content ranging from 34.90% to 36.11%, confirming a fat reduction exceeding 30% compared to standard formulations and qualifying for the “reduced fat” claim under international regulations [99]. Simultaneously, the TDF content in the cookies ranged from 10.03% to 13.29% (Table 3). Given that the required threshold for a “high fiber” claim is 6 g/100 g, all formulations easily met this nutritional criterion [99,101]. The highest TDF (13.29%) was recorded in the boiling-coarse (BC) sample, driven by the inherently higher crude fiber content of the coarse DCR fractions.

A critical observation arose from the SDF analysis. The autoclaved-fine (AF) cookie exhibited the highest SDF content (1.62%), which sharply contrasts with the low SDF observed in the corresponding raw AF DCR powder. This notable reversal suggests that the thermal process of baking (150 °C for 13 min) induced further structural changes through two main mechanisms. The first was heat-induced solubilization, where baking temperatures triggered a partial conversion of the remaining IDF into SDF [102,103]. This was most effective in the autoclaved sample (AF) because the initial high-pressure treatment had already weakened the lignocellulosic structure [101]. The second was resistant starch (RS) formation, where heating and cooling during baking induced gelatinization and retrogradation of the wheat starch matrix, leading to RSIII formation [104,105]. The autoclaved fiber’s prior severe heat history likely favored significantly greater RS formation, contributing to the higher measured SDF content [65].

#### 3.2.2. Physical Attributes and Baking Loss

**Figure 3:**
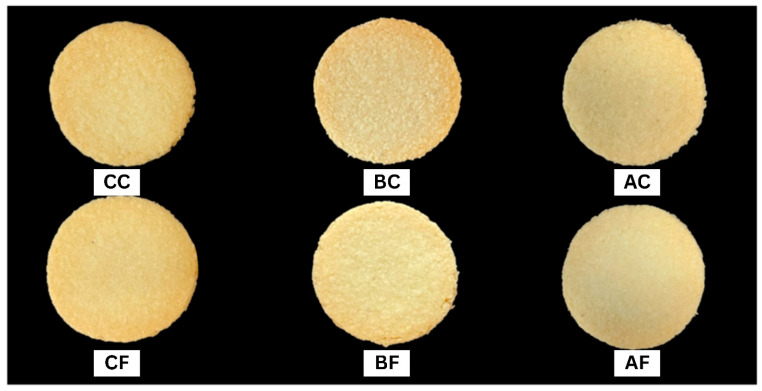
External physical appearance of reduced-fat cookies: effect of the DCR hydrothermal method and particle size on cookie geometry and surface color.

The DCR fiber significantly influenced the physical attributes of the cookies (Table 4). The untreated-coarse (CC) sample exhibited the highest spread ratio (8.65), while the fine particle size fractions (BF and AF) yielded the lowest (6.87–7.06). The visual appearance (Figure 3) confirms this trend, showing restricted flow and a thicker profile in fine-particle formulations compared to the expansive spread of the coarse control. This outcome establishes a direct inverse correlation between the DCR’s water-holding capacity (WHC) and the final spread ratio [106,107].

The fine fractions (BF and AF) actively sequestered available water in the dough, leading to “water competition” that limited the dissolution of sugar necessary to form a low-viscosity syrup phase [106,108]. Consequently, the substantial water binding increased the apparent dough viscosity, thereby restricting radial expansion during baking. Regarding moisture management, the fine fractions showed the lowest baking weight loss (8.7–8.8%), acting as moisture stabilizers; the high WHC traps water via capillary action and hydrogen bonding, limiting evaporation during baking [109,110].

#### 3.2.3. Texture and Rheology (Dough/Cookie Hardness)

Dough and final cookie hardness exhibited highly significant differences across treatments, reinforcing the role of DCR’s functional properties (Table 4). Dough hardness strongly correlated with fiber WHC and particle size. The fine fractions (BF and AF) resulted in the stiffest doughs (approx. 440 N). This is explained by the mechanisms of water competition and network disruption: high-WHC fibers absorb essential water, severely reducing the plasticizing effect of water and leading to partial dehydration of gluten and starch components [70,111]. This water sequestration increases the elastic modulus (G′) of the dough matrix, resulting in a firmer, less elastic structure [70,112]. This trend carried over to the final product, where the fine fractions produced the hardest cookies (27 N). This strong positive correlation confirms that the initial hydrocolloid water-binding capacity determines the baked product’s textural integrity.

Cookie hardness was significantly influenced by the M × P interaction, with fine fractions (BF and AF) producing the firmest texture. This can be explained by the high WHC of these modified fibers, which induces intense water competition within the dough system. High-WHC fibers sequester available water, increasing dough viscosity and rigidity, which ultimately leads to a harder baked product [113,114]. This textural change is directly linked to the fiber’s particle size and porosity, as finer particles with higher hydration capacity exacerbate the dehydration of the starch-gluten matrix during baking [14,115].

#### 3.2.4. Color and Browning Index (BI)

The final cookie color was primarily influenced by the hydrothermal treatment applied to the DCR (Table 4). The autoclaved-coarse (AC) cookie exhibited the lowest lightness (L* = 73.24) and the highest BI (52.95). Conversely, the AF cookie showed the highest lightness (L* = 76.21) among treated groups. This “BI reversal”—where the most severely heat-treated DCR did not produce the darkest cookie—confirms the success of the DCR purification and washing steps. The rigorous washing effectively removed water-soluble Maillard reaction precursors (reducing sugars and soluble proteins) from the DCR powder [23,34]. Consequently, despite the initial darkening of the raw DCR powder, browning in the final cookie was suppressed due to reduced substrate availability, resulting in a more desirable lighter color.

## 4. Conclusions

This study successfully demonstrated the potential of defatted coconut residue (DCR) powder as a high-fiber, functional fat replacer in cookie formulations. The findings established that hydrothermal treatments (boiling and autoclaving) combined with particle size modification significantly enhanced the techno-functional attributes of the residue. Specifically, the hydrothermal processes were highly effective in purifying the DCR by substantially reducing crude fat and removing soluble non-fiber components, thereby achieving a highly concentrated fiber profile with total dietary fiber (TDF) ranging from 63.77% to 73.94%. This purification process was critical for unblocking internal capillary networks and maximizing surface area, thereby significantly improving oil holding capacity (OHC) and water holding capacity (WHC). The fine particle fractions (boiling-fine [BF] and autoclaved-fine [AF]) were identified as the optimal functional ingredients, exhibiting peak WHC (10.95 g/g) and OHC (4.57 g/g).

When incorporated into the cookie formulation, all DCR treatments validated the feasibility of developing healthier baked goods, yielding products that qualify for both “reduced-fat” (30% fat reduction) and “high-fiber” (6 g/100 g) claims under international nutritional regulations. However, the high WHC of these optimal fractions directly influenced the final product quality by competing with water in the dough. This resulted in a direct inverse correlation with cookie quality, as evidenced by a restricted spread ratio (6.87–7.06) and increased dough and cookie hardness. Furthermore, the DCR purification process enhanced color stability; the thermal leaching of Maillard precursors effectively suppressed excessive browning during baking, resulting in lighter, more desirable cookies.

In conclusion, the boiling-fine (BF) fraction offers the best balance of functional enhancement and processing simplicity for DCR valorization. To translate these findings into broad industrial applications, future research should focus on optimizing dough rheology—such as by increasing moisture content or incorporating natural emulsifiers—to mitigate the extreme water absorption of fine DCR powder. This would further improve the textural quality and spread of the cookies while retaining the significant nutritional and sustainability benefits achieved in this study.

## Figures and Tables

**Table 1:** Compositional analysis of DCR powder: proximate composition, dietary fiber profile, and interaction effects of the hydrothermal method and particle size.

Sample	Moisture (%db)	Crude Fat (%db)	Crude Protein (%db)	Crude Fiber (%db)	Ash (%db)	Carbohydrate (%db)	Soluble Dietary Fiber (SDF) (%db)	Insoluble Dietary Fiber (IDF) (%db)	Total Dietary Fiber (TDF) (%db)
Untreated-Coarse	4.17 ± 0.01 b	24.00 ± 0.01 b	4.60 ± 0.08 a	46.42 ± 0.10 c	0.92 ± 0.02 b	24.08 ± 0.21 e	1.84 ± 0.08 b	63.75 ± 1.24 c	65.59 ± 1.16 c
Untreated-Fine	4.90 ± 0.02 a	26.53 ± 0.01 a	3.54 ± 0.04 c	36.15 ± 0.21 e	1.00 ± 0.02 a	32.83 ± 0.21 c	4.99 ± 0.14 a	58.78 ± 0.05 d	63.77 ± 0.09 c
Boiling-Coarse	1.47 ± 0.04 e	17.84 ± 0.05 e	3.96 ± 0.05 b	47.14 ± 0.04 b	0.36 ± 0.00 c	30.72 ± 0.04 d	1.44 ± 0.09 c	72.50 ± 0.58 a	73.94 ± 0.49 a
Boiling-Fine	2.10 ± 0.01 d	18.92 ± 0.15 d	3.03 ± 0.04 d	41.76 ± 0.23 d	0.34 ± 0.00 cd	35.96 ± 0.34 a	1.63 ± 0.00 c	71.88 ± 0.04 a	73.51 ± 0.04 a
Autoclaved-Coarse	3.32 ± 0.02 c	17.80 ± 0.16 e	3.99 ± 0.01 b	53.72 ± 0.13 a	0.31 ± 0.01 e	24.21 ± 0.01 e	1.21 ± 0.06 d	71.86 ± 1.86 a	73.07 ± 1.80 ab
Autoclaved-Fine	2.12 ± 0.04 d	19.87 ± 0.06 c	2.61 ± 0.04 e	42.11 ± 0.12 d	0.31 ± 0.00 de	35.11 ± 0.02 b	1.86 ± 0.03 b	69.37 ± 0.03 b	71.23 ± 0.00 b
Hydrothermal Method (M)	<0.001 *	<0.001 *	<0.001 *	<0.001 *	<0.001 *	<0.001 *	<0.001 *	<0.001 *	<0.001 *
Particle size (P)	0.015 *	<0.001 *	<0.001 *	0.025 *	<0.001 *	<0.001 *	<0.001 *	0.003 *	0.039 *
Interaction (M × P)	<0.001 *	<0.001 *	0.002 *	0.003 *	<0.001 *	<0.001 *	<0.001 *	0.046 *	NS

Carbohydrate was calculated by difference: 100 − (Moisture + Fat + Protein + Ash + Crude Fiber). Total Dietary Fiber (TDF) was calculated as: TDF = SDF + IDF. Means within a column with different lowercase letters (a–e) are significantly different (*p* < 0.05) based on Tukey’s multiple range test. * Significant effect at *p* < 0.05; NS, not significant at *p* < 0.05.

**Table 2:** Functional and optical properties of DCR powder: water/oil holding capacity, water solubility index, color parameters, and interaction effects of the hydrothermal method and particle size.

Sample	Water Holding Capacity (WHC) (g/g)	Water Solubility Index (WSI) (g/g)	Oil Holding Capacity (OHC) (g/g)	L*	a*	b*	Whiteness Index (WI)
Untreated-Coarse	8.00 ± 0.04 e	3.43 ± 0.07 b	3.39 ± 0.04 e	84.63 ± 0.06 b	0.22 ± 0.04 c	8.93 ± 0.06 b	82.23 ± 0.08 b
Untreated-Fine	9.59 ± 0.11 b	3.86 ± 0.08 a	3.93 ± 0.06 d	86.78 ± 0.11 a	0.22 ± 0.01 c	7.81 ± 0.01 d	84.64 ± 0.10 a
Boiling-Coarse	9.12 ± 0.16 c	1.10 ± 0.06 c	3.94 ± 0.01 d	79.53 ± 0.19 e	0.90 ± 0.02 b	8.26 ± 0.08 c	77.91 ± 0.21 d
Boiling-Fine	10.95 ± 0.13 a	1.17 ± 0.05 c	4.57 ± 0.01 a	80.81 ± 0.11 d	0.08 ± 0.01 d	7.90 ± 0.16 d	79.24 ± 0.04 c
Autoclaved-Coarse	8.55 ± 0.06 d	1.14 ± 0.16 c	4.09 ± 0.08 c	75.65 ± 0.04 f	1.18 ± 0.00 a	9.13 ± 0.03 a	73.96 ± 0.04 e
Autoclaved-Fine	10.95 ± 0.03 a	1.13 ± 0.06 c	4.35 ± 0.05 b	83.78 ± 0.03 c	0.06 ± 0.01 d	6.73 ± 0.01 e	82.44 ± 0.02 b
Hydrothermal Method (M)	<0.001 *	<0.001 *	<0.001 *	<0.001 *	<0.001 *	0.001 *	<0.001 *
Particle size (P)	<0.001 *	0.018 *	<0.001 *	<0.001 *	<0.001 *	<0.001 *	<0.001 *
Interaction (M × P)	0.003 *	0.026 *	0.004 *	<0.001 *	<0.001 *	<0.001 *	<0.001 *

Means within a column with different lowercase letters (a–f) are significantly different (*p* < 0.05) based on Tukey’s multiple range test. * Significant effect at *p* < 0.05. L*: lightness; a*: redness/greenness; b*: yellowness/blueness.

**Table 3:** Proximate and dietary fiber composition of reduced-fat cookies: interaction effects of the DCR hydrothermal method and particle size.

Sample Formulation	Moisture (%db)	Crude Fat (%db)	Crude Protein (%db)	Crude Fiber (%db)	Ash (%db)	Carbohydrate (%db)	Soluble Dietary Fiber (SDF) (%db)	Insoluble Dietary Fiber (IDF) (%db)	Total Dietary Fiber (TDF) (%db)
Untreated-Coarse	2.26 ± 0.01 a	36.05 ± 0.26 a	5.31 ± 0.04 a	6.95 ± 0.19 e	0.33 ± 0.01 ab	51.37 ± 0.04 c	1.40 ± 0.08 b	9.25 ± 0.02 d	10.64 ± 0.06 d
Untreated-Fine	1.96 ± 0.05 c	36.11 ± 0.06 a	5.04 ± 0.03 b	5.91 ± 0.04 f	0.33 ± 0.04 a	52.64 ± 0.03 a	1.13 ± 0.00 c	8.90 ± 0.16 d	10.03 ± 0.16 e
Boiling-Coarse	2.09 ± 0.01 b	34.91 ± 0.03 b	4.96 ± 0.04 b	8.56 ± 0.07 b	0.27 ± 0.00 c	51.30 ± 0.07 c	1.37 ± 0.09 b	11.92 ± 0.00 a	13.29 ± 0.09 a
Boiling-Fine	2.24 ± 0.01 a	35.06 ± 0.17 b	4.81 ± 0.04 c	7.84 ± 0.01 c	0.28 ± 0.01 bc	51.96 ± 0.05 b	0.92 ± 0.03 d	11.10 ± 0.34 b	12.02 ± 0.31 c
Autoclaved-Coarse	2.13 ± 0.01 b	34.90 ± 0.12 b	4.96 ± 0.06 b	10.21 ± 0.07 a	0.26 ± 0.01 c	49.76 ± 0.01 d	0.85 ± 0.03 d	11.58 ± 0.09 a	12.43 ± 0.06 b
Autoclaved-Fine	1.86 ± 0.01 d	35.03 ± 0.09 b	4.77 ± 0.01 c	7.44 ± 0.13 d	0.24 ± 0.01 c	52.56 ± 0.10 a	1.62 ± 0.07 a	10.50 ± 0.08 c	12.12 ± 0.01 bc
Hydrothermal Method (M)	<0.001 *	<0.001 *	<0.001 *	<0.001 *	0.003 *	<0.001 *	NS	<0.001 *	<0.001 *
Particle size (P)	<0.001 *	NS	<0.001 *	<0.001 *	NS	<0.001 *	NS	<0.001 *	<0.001 *
Interaction (M × P)	<0.001 *	NS	NS	<0.001 *	NS	<0.001 *	<0.001 *	NS	0.011 *

Carbohydrate was calculated by difference: 100 − (Moisture + Fat + Protein + Ash + Crude Fiber). Total Dietary Fiber (TDF) was calculated as TDF = SDF + IDF. Means within a column with different lowercase letters (a–f) are significantly different (*p* < 0.05) based on Tukey’s HSD post hoc test. * Significant effect at *p* < 0.05; NS, not significant at *p* < 0.05.

**Table 4:** Physical, textural, and color attributes of reduced-fat cookies: interaction effects of the DCR hydrothermal method and particle size.

Sample Formulation	Diameter (mm)	Thickness (mm)	Spread Ratio	Baking Weight Loss (%)	Dough Hardness (N)	Baked Cookie Hardness (N)	L*	a*	b*	Browning Index
Untreated-Coarse	41.11 ± 0.22 a	4.76 ± 0.20 d	8.65 ± 0.33 a	10.93 ± 0.33 a	153.91 ± 2.25 e	11.79 ± 0.77 a	73.99 ± 0.55 d	5.04 ± 0.14 ab	29.16 ± 0.48 a	52.95 ± 0.64 a
Untreated-Fine	40.08 ± 0.37 b	5.15 ± 0.15 c	7.65 ± 0.27 b	10.78 ± 0.19 a	271.97 ± 5.91 c	18.42 ± 0.97 c	74.85 ± 0.38 c	5.36 ± 0.22 a	28.65 ± 0.39 b	52.07 ± 0.64 a
Boiling-Coarse	40.09 ± 0.11 b	5.30 ± 0.16 bc	7.57 ± 0.21 b	9.73 ± 0.14 b	324.53 ± 4.30 b	18.02 ± 0.78 c	75.46 ± 0.59 b	4.67 ± 0.42 bc	27.86 ± 0.41 c	49.18 ± 0.61 b
Boiling-Fine	38.01 ± 0.14 d	5.54 ± 0.17 a	6.87 ± 0.22 c	8.69 ± 0.12 c	436.25 ± 7.63 a	27.70 ± 0.95 a	76.32 ± 0.48 a	4.58 ± 0.25 c	27.62 ± 0.17 c	48.35 ± 0.77 b
Autoclaved-Coarse	39.89 ± 0.27 b	5.36 ± 0.12 abc	7.41 ± 0.13 b	9.41 ± 0.39 b	261.96 ± 2.70 d	20.35 ± 0.70 b	73.24 ± 0.23 e	4.29 ± 0.38 c	27.05 ± 0.42 d	49.37 ± 0.82 b
Autoclaved-Fine	38.93 ± 0.20 c	5.51 ± 0.13 ab	7.06 ± 0.17 c	8.84 ± 0.19 c	441.86 ± 6.93 a	27.27 ± 1.42 a	76.21 ± 0.29 a	4.31 ± 0.40 c	26.60 ± 0.35 d	46.17 ± 0.70 c
Hydrothermal Method (M)	<0.001 *	<0.001 *	<0.001 *	<0.001 *	<0.001 *	<0.001 *	<0.001 *	<0.001 *	<0.001 *	<0.001 *
Particle size (P)	<0.001 *	<0.001 *	<0.001 *	<0.001 *	<0.001 *	<0.001 *	<0.001 *	NS	0.009 *	<0.001 *
Interaction (M × P)	NS	<0.001 *	0.015 *	0.002 *	<0.001 *	0.003 *	<0.001 *	NS	NS	0.003 *

Spread ratio was calculated as the ratio of cookie diameter to thickness (Spread Ratio = Diameter/Thickness). Means within a column with different lowercase letters (a–e) are significantly different (*p* < 0.05) based on Tukey’s HSD post hoc test. * Significant effect at *p* < 0.05; NS, not significant at *p* < 0.05. L*: lightness; a*: redness/greenness; b*: yellowness/blueness.

## Data Availability

The original contributions presented in this study are included in the article. Further inquiries can be directed to the corresponding author.

## References

[1] Tapia-Quirós P., Montenegro-Landívar M.F., Vecino X., Alvarino T., Cortina J.L., Saurina J., Granados M., Reig M. (2022). A green approach to phenolic compounds recovery from olive mill and winery wastes. Sci. Total Environ..

[2] Esposito T., Silva N.H.C.S.S., Almeida A., Silvestre A.J.D., Piccinelli A., Aquino R.P., Sansone F., Mencherini T., Vilela C., Freire C.S.R. (2020). Valorisation of chestnut spiny burs and roasted hazelnut skins extracts as bioactive additives for packaging films. Ind. Crops Prod..

[3] Khalid L., Jabeen I., Ahmad T., Inam-ur-Raheem M., Shahid A., Cirillo T., Esposito F. (2025). Valorization of fruit and vegetable by-products for protein extraction and their functional applications in food and non-food sectors. Food Bioprod. Process..

[4] Camacho M.d.M., Fernández-Vargas N., García-Martínez E., Martínez-Navarrete N. (2025). Influence of the use of gum Arabic or OSA starch and the drying process on the quality of the fava bean (*Vicia faba*) pod flour. Food Hydrocoll. Health.

[5] Lomba-Viana X., Raymundo A., Prista C., Alegria M.J., Sousa I. (2022). Clean Label “Rocha” Pear (*Pyrus communis* L.) Snack Containing Juice By-Products and Euglena gracilis Microalgae. Front. Nutr..

[6] Tin K.K., Taweepreda W., Kumar A. (2025). Current trends and future prospects of hydrogen production from coconut waste. Int. J. Hydrogen Energy.

[7] Sopandi T.P., Sulianto A.A., Anugroho F., Yusoff M.Z.M., Mohamed M.S., Farid M.A.A., Setyawan H.Y. (2025). RSM-optimized biochar production from young coconut waste (*Cocos nucifera*): Multivariate analysis of non-linear interactions between temperature, time, and activator concentration. Ind. Crops Prod..

[8] Salaenoi J., Jurejan N., Yokthongwattana C., Pluempanupat W., Boonprab K. (2024). Characteristics of coconut husk cellulose and its effectiveness as a potassium permanganate absorbent for fishery applications. Case Stud. Chem. Environ. Eng..

[9] Manatura K., Samsalee N., Kaewtrakulchai N., Jadsadajerm S., Muangklang E., Jaruwongwittaya T., Huang C.-W. (2025). Optimization of torrefaction parameters for coconut shell using Taguchi method: Impact on torrefaction performances, combustion characteristics, and thermal stability. Therm. Sci. Eng. Prog..

[10] Socas-Rodríguez B., Álvarez-Rivera G., Valdés A., Ibáñez E., Cifuentes A. (2021). Food by-products and food wastes: Are they safe enough for their valorization?. Trends Food Sci. Technol..

[11] Ceballos M.W., Jafari S., Fikry M., Shiekh K.A., Kijpatanasilp I., Assatarakul K. (2025). Changes in quality attributes of coconut water treated with UV-radiation and nisin during cold storage: Kinetics modelling and shelf-life prediction. Food Control.

[12] Du X., Wang L., Huang X., Jing H., Ye X., Gao W., Bai X., Wang H. (2021). Effects of different extraction methods on structure and properties of soluble dietary fiber from defatted coconut flour. LWT.

[13] Rodsamran P., Sothornvit R. (2018). Physicochemical and functional properties of protein concentrate from by-product of coconut processing. Food Chem..

[14] Shakeela H., Mohan K., Nisha P. (2024). Unlocking a nutritional treasure: Health benefits and sustainable applications of spent coconut meal. Sustain. Food Technol..

[15] Liu X.-Y., Yang D.-W., Liu W.-T., Kan J.-T., Yang K.-L., Zhang J.-G., Wang Y.-Y., Zhu K.-X., Zhang Y.-F. (2025). Structural and techno-functional characteristics of protein from the by-product of virgin coconut oil produced by centrifugation using coconut milk. Food Chem. X.

[16] Raghavendra S.N., Ramachandra Swamy S.R., Rastogi N.K., Raghavarao K.S.M.S., Kumar S., Tharanathan R.N. (2006). Grinding characteristics and hydration properties of coconut residue: A source of dietary fiber. J. Food Eng..

[17] Yalegama L.L.W.C., Karunaratne D.N., Sivakanesan R., Jayasekara C. (2013). Chemical and functional properties of fibre concentrates obtained from by-products of coconut kernel. Food Chem..

[18] Gao X., Pei Z., Yi X., Zhang X., He D., Feng Z., Xia G., Shen X. (2024). Development and characterization of defatted coconut flour based oleogels: A fat substitute for application in oil-fortified surimi. Food Chem. X.

[19] Vishwakarma S., Dalbhagat C.G., Mandliya S., Mishra H.N. (2022). Investigation of natural food fortificants for improving various properties of fortified foods: A review. Food Res. Int..

[20] Adeloye J.B., Osho H., Idris L.O. (2020). Defatted coconut flour improved the bioactive components, dietary fibre, antioxidant and sensory properties of nixtamalized maize flour. J. Agric. Food Res..

[21] Wei C., Ge Y., Liu D., Zhao S., Wei M., Jiliu J., Hu X., Quan Z., Wu Y., Su Y. (2021). Effects of High-Temperature, High-Pressure, and Ultrasonic Treatment on the Physicochemical Properties and Structure of Soluble Dietary Fibers of Millet Bran. Front. Nutr..

[22] Zhang Y., Qi J., Zeng W., Huang Y., Yang X. (2020). Properties of dietary fiber from citrus obtained through alkaline hydrogen peroxide treatment and homogenization treatment. Food Chem..

[23] Bocker R., Silva E.K. (2026). Electric field-based technologies for functional modification of food biopolymers: Recent advances and trends in proteins, dietary fibers and starches. Trends Food Sci. Technol..

[24] Phùng T.-T., Đinh H.-N., Ureña M., Oliete B., Denimal E., Dupont S., Beney L., Karbowiak T. (2025). Sodium Alginate as a promising encapsulating material for extremely-oxygen sensitive probiotics. Food Hydrocoll..

[25] Menon L., Sanjanwala D., Sharma S., Parul, Jain R., Dandekar P. (2025). Sterilizing bioinks: Understanding the impact of techniques on 3D bioprinting materials. Bioprinting.

[26] Tappiban P., Sraphet S., Srisawad N., Ahmed S., Bao J., Triwitayakorn K. (2024). Cutting-edge progress in green technologies for resistant starch type 3 and type 5 preparation: An updated review. Food Chem. X.

[27] Nedeljković N., Hadnađev M., Dapčević Hadnađev T., Šarić B., Pezo L., Sakač M., Pajin B. (2017). Partial replacement of fat with oat and wheat bran gels: Optimization study based on rheological and textural properties. LWT.

[28] Steiner J., Franke K., Kießling M., Fischer S., Töpfl S., Heinz V., Becker T. (2018). Influence of hydrothermal treatment on the structural modification of spent grain specific carbohydrates and the formation of degradation products using model compounds. Carbohydr. Polym..

[29] Bucsella B., Takács Á., Vizer V., Schwendener U., Tömösközi S. (2016). Comparison of the effects of different heat treatment processes on rheological properties of cake and bread wheat flours. Food Chem..

[30] Ren M., Hou Y., Peng D., Li H., Zhang X., Qiao L., Wang X., Jiang Y., Wu F., Wang G. (2025). Ultrasonic/compound enzyme extraction, comparative characterisation and biological activity of Lonicera macranthoides polysaccharides. Ultrason. Sonochem..

[31] Huang D., Xu Y., Zhang W., Liu Y., Zhang T., Liu H., Jiang Y., Li D. (2024). Enhancement of foaming property of ormosia protein: Insights into the effect of high-intensity ultrasound on physicochemical properties and structure analysis. Food Hydrocoll..

[32] Wang J., Gan H., Tang Y., He H., Sun M., Chen Y., Deng Q., Huang F., Tang H. (2025). Effect of particle size of Flaxseed-based milk coproduct on the quality of dough and steamed bread. Oil Crop Sci..

[33] Xu M., Qi M., Goff H.D., Cui S.W. (2020). Polysaccharides from sunflower stalk pith: Chemical, structural and functional characterization. Food Hydrocoll..

[34] Gamonpilas C., Buathongjan C., Kirdsawasd T., Rattanaprasert M., Klomtun M., Phonsatta N., Methacanon P. (2021). Pomelo pectin and fiber: Some perspectives and applications in food industry. Food Hydrocoll..

[35] Jacob J., Leelavathi K. (2007). Effect of fat-type on cookie dough and cookie quality. J. Food Eng..

[36] Kouhsari F., Saberi F., Kowalczewski P.Ł., Lorenzo J.M., Kieliszek M. (2022). Effect of the various fats on the structural characteristics of the hard dough biscuit. LWT.

[37] Sanz T., Salvador A., Hernández M.J., Ahmed J., Basu S. (2023). Creep–recovery and oscillatory rheology of flour-based systems. Advances in Food Rheology and Its Applications.

[38] Manley D. (2000). Fats and oils. Technology of Biscuits, Crackers and Cookies.

[39] Gallagher E., Gallagher E. (2008). Formulation and nutritional aspects of gluten-free cereal products and infant foods. Gluten-Free Cereal Products and Beverages.

[40] Davidson I. (2025). Ingredients for biscuits: An introduction. Biscuit, Cookie and Cracker Production.

[41] Atkinson G., Talbot G. (2011). Saturated fat reduction in biscuits. Reducing Saturated Fats in Foods.

[42] Bhat N.A., Wani I.A., Hamdani A.M. (2020). Tomato powder and crude lycopene as a source of natural antioxidants in whole wheat flour cookies. Heliyon.

[43] Mert B., Demirkesen I. (2016). Evaluation of highly unsaturated oleogels as shortening replacer in a short dough product. LWT-Food Sci. Technol..

[44] Li S., Wu G., Li X., Jin Q., Wang X., Zhang H. (2021). Roles of gelator type and gelation technology on texture and sensory properties of cookies prepared with oleogels. Food Chem..

[45] Subhasri D., Dutta S., Leena M.M., Moses J.A., Anandharamakrishnan C. (2022). Gastronomy: An extended platform for customized nutrition. Future Foods.

[46] Genovese A., Balivo A., Salvati A., Sacchi R. (2022). Functional ice cream health benefits and sensory implications. Food Res. Int..

[47] O’Sullivan M.G. (2020). Reduced-fat products and challenges. Salt, Fat and Sugar Reduction: Sensory Approaches for Nutritional Reformulation of Foods and Beverages.

[48] Zoulias E.I., Oreopoulou V., Tzia C. (2002). Textural properties of low-fat cookies containing carbohydrate- or protein-based fat replacers. J. Food Eng..

[49] Milićević N., Sakač M., Hadnađev M., Škrobot D., Šarić B., Dapčević Hadnađev T., Jovanov P., Pezo L. (2020). Physico-chemical properties of low-fat cookies containing wheat and oat bran gels as fat replacers. J. Cereal Sci..

[50] Soares I.D., Junqueira I.G., Cirilo M.E.M., Vanin F.M., Rodrigues C.E.d.C. (2025). Defatted cocoa bean shells as an alternative flour for cookie production within a circular economy approach. Food Res. Int..

[51] Wang C.-C., Yang Z., Xing J.-J., Guo X.-N., Zhu K.-X. (2021). Effects of insoluble dietary fiber and ferulic acid on the rheological properties of dough. Food Hydrocoll..

[52] Cui S.W., Nie S., Roberts K.T. (2011). Functional Properties of Dietary Fiber. Comprehensive Biotechnology.

[53] Yavuz-Düzgün M., Kirkin C., Ozkan G. (2025). Physicochemical attributes of irradiated proteins. Non-Thermal Processing of Major Food Macromolecules.

[54] Kadam D.M., Parab S.S., Kasara A., Dange M.M., Mahawar M.K., Kumar M., Arude V.G. (2023). Effect of microwave pre-treatment on protein extraction from de-oiled cottonseed meal and its functional and antioxidant properties. Food Humanit..

[55] Çiçek S., Işık S. (2025). Development of functional bread and other bakery products. Handbook of Sourdough Microbiota and Fermentation.

[56] Guo X., Dai T., Chen M., Deng L., Chen J., Liu C. (2022). Steam bread made by superfine purple corn flour: Texture characteristics and in vitro starch digestibility. LWT.

[57] Mariod A.A. (2018). Gum Arabic Dietary Fiber. Gum Arabic: Structure, Properties, Application and Economics.

[58] Muala W.C.B., Charnelle T.K., Fabrice T.D., Bernard T., Ghislain M.N., Serge N.E. (2024). Formulation of weaning food from yellow maize (*Zea mays* L.) and red millet (*Eleusine coracana* L.), enriched with pretreated African locust beans (*Parkia biglobosa* Jacq.) flour. J. Agric. Food Res..

[59] Lin S. (2022). Dietary fiber in bakery products: Source, processing, and function. Adv. Food Nutr. Res..

[60] Jie S., Zheng C., Hao W., Linkun S., Yizhuo L. (2025). Evaluation on performance and carbon-sequestration of spent coffee grounds hydrothermal biochar concrete under multi-factor interaction. Constr. Build. Mater..

[61] Nam H.-J., Ishii R., Ebina T., Mizukami F. (2009). Flexible transparent self-standing binderless clay film prepared by hydrothermally-treated synthetic clay. Mater. Lett..

[62] Laguna L., Salvador A., Sanz T., Fiszman S.M. (2011). Performance of a resistant starch rich ingredient in the baking and eating quality of short-dough biscuits. LWT.

[63] Blanco Canalis M.S., Baroni M.V., León A.E., Ribotta P.D. (2020). Effect of peach puree incorporation on cookie quality and on simulated digestion of polyphenols and antioxidant properties. Food Chem..

[64] Marshall R.T. (2003). ICE CREAM | Properties and Analysis. Encyclopedia of Food Sciences and Nutrition.

[65] Kurian S., Mounika A., Rao T.J., Sherman I.M., Jayakumar J., Muralitharan J., Shanmugam A. (2025). Comparative study between conventional and ultrasonic homogenization techniques on the formulated vegan frozen dessert made using chickpea and mung bean protein isolates. Food Chem. Adv..

[66] Kashaninejad M., Razavi S.M.A. (2020). Influence of thermosonication treatment on the average size of fat globules, emulsion stability, rheological properties and color of camel milk cream. LWT.

[67] Jumaa M., Müller B.W. (1999). Physicochemical properties of chitosan-lipid emulsions and their stability during the autoclaving process. Int. J. Pharm..

[68] Ranasinghe M., Stathopoulos C., Sundarakani B., Maqsood S. (2024). Valorizing date seeds through ultrasonication to enhance quality attributes of dough and biscuit, Part-1: Effects on dough rheology and physical properties of biscuits. Ultrason. Sonochem..

[69] Mahajan P., Bera M.B., Panesar P.S. (2022). Structural, functional, textural characterization and in vitro digestibility of underutilized Kutki millet (*Panicum sumatrense*) starch. LWT.

[70] Hort J., Cook D. (2007). Formulating low-fat food: The challenge of retaining flavour quality. Modifying Flavour in Food.

[71] Association of Official Analytical Chemists (2019). Official Methods of Analysis of AOAC International.

[72] Sorde K.L., Ananthanarayan L. (2019). Effect of transglutaminase treatment on properties of coconut protein-guar gum composite film. LWT.

[73] AACC International (2009). Determination of Baking Quality of Cookie Flour—Simplified Wire-Cut Formula. Approved Methods of Analysis.

[74] Bernaś E., Jaworska G. (2015). Nutritional and non-nutritional compounds of *Agaricus bisporus* during thermal processing. LWT.

[75] Ayvaz H., Korkmaz F., Polat H., Ayvaz Z., Tuncel N.B. (2021). Detection of einkorn flour adulteration in flour and bread samples using Computer-Based Image Analysis and Near-Infrared Spectroscopy. Food Control.

[76] Park J., Choi I., Kim Y. (2015). Cookies formulated from fresh okara using starch, soy flour and hydroxypropyl methylcellulose have high quality and nutritional value. LWT-Food Sci. Technol..

[77] Mudgil D., Barak S., Khatkar B.S. (2017). Cookie texture, spread ratio and sensory acceptability of cookies as a function of soluble dietary fiber, baking time and different water levels. LWT-Food Sci. Technol..

[78] Hanafi F.N.A., Kamaruding N.A., Shaharuddin S. (2022). Influence of coconut residue dietary fiber on physicochemical, probiotic (*Lactobacillus plantarum* ATCC 8014) survivability and sensory attributes of probiotic ice cream. LWT.

[79] Bayram M., Kaya A., Öner M.D. (2004). Changes in properties of soaking water during production of soy-bulgur. J. Food Eng..

[80] Tiruta-Barna L., Barna R. (2012). Potential hazards from waste based/recycled building materials. Toxicity of Building Materials.

[81] Yan Y., Zhou S., Tang S., Wang N., Gao X., Wang Y., Zhu H., Li Z., Sun H. (2025). Effect of different particle size on microstructure, hydration characteristics, and flow properties of bulb powders from Lanzhou Lily (*Lilium davidii* var. unicolor). Food Struct..

[82] Wennberg M., Nyman M. (2004). On the possibility of using high pressure treatment to modify physico-chemical properties of dietary fibre in white cabbage (*Brassica oleracea* var. capitata). Innov. Food Sci. Emerg. Technol..

[83] Palencia M., Chate N.G., García-Quintero A. (2024). Obtaining micrometric-scale hollow fibers from wastes aged coir by oxidative chloro-sulfonation of lignin. Bioresour. Technol. Rep..

[84] Yin L., Chen Y., Li D., Zhao X., Hou B., Cao B. (2016). 3-Dimensional hierarchical porous activated carbon derived from coconut fibers with high-rate performance for symmetric supercapacitors. Mater. Des..

[85] Rovaris Â.A., Dias C.O., da Cunha I.P., Scaff R.M.C., de Francisco A., Petkowicz C.L., Amante E.R. (2012). Chemical composition of solid waste and effect of enzymatic oil extraction on the microstructure of soybean (*Glycine max*). Ind. Crops Prod..

[86] Siah S., Wood J.A., Agboola S., Konczak I., Blanchard C.L. (2014). Effects of soaking, boiling and autoclaving on the phenolic contents and antioxidant activities of faba beans. Food Chem..

[87] Zou Y., Bi J., Liu X., Yi J., Lyu J., Peng J., Wu X. (2017). Physicochemical and functional properties of dietary fiber-rich powders derived from asparagus (*Asparagus officinalis* L.) stem by-product as affected by different preparation methods. LWT.

[88] Lin Q., Yang X., Ma W., Zahoor A., Jin F., Chen X., Tai L., de Caprariis B., De Filippis P., Damizia M. (2025). Hydrothermal carbonization of fish sludge: Effect of FeCl_3_ on the hydrochar properties. Environ. Technol. Innov..

[89] Martínez R., Torres P., Meneses M.A., Figueroa J.G., Pérez-Álvarez J.A., Viuda-Martos M. (2012). Chemical, technological and in vitro antioxidant properties of mango, guava, pineapple and passion fruit dietary fibre concentrate. Food Chem..

[90] Marler T.E., Lindström A.J. (2014). Free sugar profile in cycads. Front. Plant Sci..

[91] Ullah I., Yin T., Xiong S., Huang Q., Din Z.U., Zhang J., Javaid A.B. (2018). Effects of thermal pre-treatment on physicochemical properties of nano-sized okara insoluble dietary fiber prepared by wet media milling. J. Food Eng..

[92] Nyman M., Haskå L., Delcour J.A., Poutanen K. (2013). Vegetable, fruit and potato fibres. Fibre-Rich and Wholegrain Foods: Improving Quality.

[93] Resende L.M., Franca A.S., Oliveira L.S. (2019). Buriti (*Mauritia flexuosa* L. f.) fruit by-products flours: Evaluation as source of dietary fibers and natural antioxidants. Food Chem..

[94] Van Audenhove J., Bernaerts T., Putri N.I., Van Rooy L., Van Loey A.M., Hendrickx M.E. (2022). The role of mechanical collapse by cryogenic ball milling on the effect of high-pressure homogenization on the microstructural and texturizing properties of partially pectin-depleted tomato cell wall material. Food Res. Int..

[95] Zhu H., Li H.-T., Fan X., Wang T., Dhital S. (2025). Modification of cellulosic structures from fruit by-products: Toward better nutritional properties. Food Res. Int..

[96] Sayem A.S.M., Talukder S., Akter S.S., Alam M., Rana R., Alam M. (2024). Utilization of fruits and vegetables wastes for the dietary fiber enrichment of biscuits and its quality attributes. J. Agric. Food Res..

[97] Ratnaningsih N., Arovah N.I. (2025). Structural, physicochemical properties, and resistant starch content of autoclaved arenga starch. Food Chem. Adv..

[98] Stevenson M., Long J., Seyfoddin A., Guerrero P., de la Caba K., Etxabide A. (2020). Characterization of ribose-induced crosslinking extension in gelatin films. Food Hydrocoll..

[99] Zotti-Sperotto N.C., de Ávila M.B., de Souza R.A., Melo E.d.C., Governici J.L., Gonzaga D.A., Fonseca M.C., Carneiro A.P.d.S., Demuner A.J., Pinheiro P.F. (2021). Intermittent drying of *Lippia origanoides* leaves and Schinus terebinthifolius fruits. Ind. Crops Prod..

[100] Afifah D.N., Ningrum Y.P.A., Syahidah T., Nuryanto N., Ayustaningwarno F., Sugianto D.N. (2022). Nutrient Content, Organoleptic Quality, and Shelf Life of Sagon Substitute from Lindur (*Bruguiera gymnorrhiza* L.) and Soybean Flour (*Glycine max* L.). Front. Nutr..

[101] Was-Gubala J. (2013). Textile and Fiber Damage. Encyclopedia of Forensic Sciences.

[102] Helou C., Jacolot P., Niquet-Léridon C., Gadonna-Widehem P., Tessier F.J. (2016). Maillard reaction products in bread: A novel semi-quantitative method for evaluating melanoidins in bread. Food Chem..

[103] Koletta P., Irakli M., Papageorgiou M., Skendi A. (2014). Physicochemical and technological properties of highly enriched wheat breads with wholegrain non wheat flours. J. Cereal Sci..

[104] Raungrusmee S., Shrestha S., Sadiq M.B., Anal A.K. (2020). Influence of resistant starch, xanthan gum, inulin and defatted rice bran on the properties of low glycemic gluten-free noodles. LWT.

[105] Niba L.L. (2003). Effect of storage period and temperature on resistant starch and β-glucan content in cornbread. Food Chem..

[106] Rafique H.S., Hussain A., Nadeem M., Rehman A., Siddique T., Najam A., Haroon H., Arif M.R., Yaqub S., Fatima H. (2023). Impact of different proportions of sweet potato (*Ipomoea batatas* L.) flour on physical, chemical and sensory parameters of cake rusk. Food Humanit..

[107] Singh G.D., Riar C.S., Saini C., Bawa A.S., Sogi D.S., Saxena D.C. (2011). Indian water chestnut flour: Method optimization, physicochemical properties and potential in cookies preparation. LWT.

[108] Kahraman K., Aktas-Akyildiz E., Ozturk S., Koksel H. (2019). Effect of different resistant starch sources and wheat bran on dietary fibre content and in vitro glycaemic index of cookies. J. Cereal Sci..

[109] Sun H., Ma J., Cao Q., Ren G., Li Z., Xie H., Huang M. (2024). Seaweed soluble dietary fibre replacement modulates the metabolite release of cakes after in vitro digestion. Int. J. Biol. Macromol..

[110] Phongthai S., D’amico S., Schoenlechner R., Rawdkuen S. (2016). Comparative study of rice bran protein concentrate and egg albumin on gluten-free bread properties. J. Cereal Sci..

[111] Li M., Zheng X., Sun B., Li L., Wang X., Ma S. (2025). Effects of the interaction of different particle size wheat bran dietary fiber with gluten protein and starch on dough structure. Int. J. Biol. Macromol..

[112] Sadeghi A., Kardooni Z., Ebrahimi M., Assadpour E., Jafari S.M. (2025). The role of water alternatives in bread formulation and its quality. Future Foods.

[113] Struck S., Rohm H., Galanakis C.M. (2020). Fruit processing by-products as food ingredients. Valorization of Fruit Processing By-Products.

[114] Vázquez-Ovando A., Rosado-Rubio G., Chel-Guerrero L., Betancur-Ancona D. (2009). Physicochemical properties of a fibrous fraction from chia (*Salvia hispanica* L.). LWT-Food Sci. Technol..

[115] Elleuch M., Bedigian D., Roiseux O., Besbes S., Blecker C., Attia H. (2011). Dietary fibre and fibre-rich by-products of food processing: Characterisation, technological functionality and commercial applications: A review. Food Chem..

